# An explainable online frailty prediction model for community-dwelling older adults based on machine learning algorithms: a cross-sectional study based on retrospective health data

**DOI:** 10.1080/07853890.2026.2647569

**Published:** 2026-04-02

**Authors:** Shuangye Zhao, Siyu Zhang, Jiawen Wang, Tinghui Huang, Huiling Wu, Weifeng Yao, Kaizong Huang, Jianjun Zou, Yuying Shen

**Affiliations:** ^a^ Department of General Practice, Nanjing First Hospital, Nanjing Medical University, Nanjing, China; ^b^ School of Basic Medicine and Clinical Pharmacy, China Pharmaceutical University, Nanjing, China; ^c^ Department of Pharmacy, Nanjing First Hospital, China Pharmaceutical University, Nanjing, China; ^d^ Jiangsu Key Laboratory for High Technology Research of TCM Formulae, National and Local Collaborative Engineering Center of Chinese Medicinal Resources Industrialization and Formulae Innovative Medicine and Jiangsu Collaborative Innovation Center of Chinese Medicinal Resources Industrialization, Nanjing University of Chinese Medicine, Nanjing, China; ^e^ Nanjing University of Chinese Medicine Hanlin College, Taizhou Key Laboratory of Traditional Chinese Medicine and Comprehensive Health Products Development, Taizhou Engineering Research Center for Quality and Industrialization of Traditional Chinese Medicine, Taizhou, China; ^f^ Department of Clinical Pharmacology Lab, Nanjing First Hospital, Nanjing Medical University, Nanjing, China

**Keywords:** Community, Frailty, Older adults, Machine learning, Prediction model

## Abstract

**Background:**

Frailty is a significant health concern associated with diminished physiological reserves and increased healthcare burdens. Currently, effective models for predicting frailty risk are lacking. This study utilizes machine learning-based models to early identify community-dwelling older adults, enhancing risk assessment accuracy and guiding targeted interventions to slow frailty progression.

**Methods:**

A cross-sectional analysis of data from 1,156 older adults across 31 community health centers in Nanjing, conducted between January and October 2024, was performed. Independent predictors of frailty were identified using univariate analysis and the least absolute shrinkage and selection operator. The dataset was divided into 70% training and 30% testing subsets. Six machine learning (ML) models were developed and their performances compared. The SHapley Additive exPlanations (SHAP) method was applied to interpret the models, and a web-based risk calculator was created.

**Results:**

Our dataset showed that 22.3% of older adults were frail. Significant predictors of frailty were identified as age, education, medicine, vegetable, cognitive status, number of diseases, hemoglobin, total cholesterol, and neutrophil-to-lymphocyte ratio. Among the six ML models, Categorical Boosting (CatBoost) exhibited the highest performance, attaining an AUROC of 0.886 in the training set and 0.831 in the testing set.

**Conclusions:**

The developed CatBoost model and web calculator can be employed by general practitioners to proactively identify high-risk community-dwelling older adults, thereby enabling timely interventions to mitigate the progression of frailty. The tool’s simplicity and replicability effectively facilitate the promotion and management of frailty prevention within the community.

## KEY MESSAGES

- An effective model was developed to predict frailty risk in community-dwelling older adults.
- The integration of biomarkers and self-reported data enhances frailty prediction and optimizes risk-based intervention strategies.
- The CatBoost model demonstrates superior predictive performance compared to other models.
- The web-based tool integrates a frailty prediction model for proactive risk management.

## Introduction

With the intensification of population aging trends, frailty has emerged as a health concern of significant prominence [1]. Frailty is recognized as a complex geriatric syndrome, highly prevalent among older adults, and characterized by the age-related decline in physiological reserves, cognitive function, social engagement, and various other systems. The impact of this condition extends beyond individual quality of life and physical functionality, imposing a considerable burden on healthcare systems and society at large [2–7]. It is estimated that the global prevalence of frailty ranges from 12% to 24% [8]. Research indicates that frailty exhibits dynamic characteristics [9]. It has been demonstrated that early screening and timely intervention targeting risk factors can lead to preventive measures and the reversal of frailty [9–13]. Consequently, the early identification of frailty trajectories and the investigation of their potential risk factors are deemed of paramount importance.

A frailty prediction model has been developed based on an investigation of factors influencing frailty in elderly patients, effectively forecasting frailty and delineating its associated risk factors. This model thereby enables precise guidance for the formulation of intervention strategies. However, existing research in this area remains limited, particularly within the hospital settings of our country. Previous studies have primarily focused on hospitalized patients, and research on frailty among community-dwelling older adults remains exceedingly scarce [3,14]. Moreover, previous studies have predominantly relied on self-reported methods to identify the risk factors for frailty in elderly individuals, leading to a lack of objective data and an increased susceptibility to measurement errors [3,15,16].

Frailty in older adults is considered a complex condition arising from the interconnected effects of multiple factors. Traditional statistical models typically illustrate the relationship between dependent and independent variables but fail to account for the comprehensive impact of multiple factors [17,18]. Machine learning (ML), as a significant branch of artificial intelligence, has gradually entered public consciousness. It is capable of generating empirical predictive models through big data, delving into the relationships among risk factors, facilitating the selection of relevant features and reducing the number of variables, while also addressing the nonlinearity issues inherent in data generation [19]. Predictive models are being utilized by health researchers to identify frail older adults [20].

In this study, various significant subjective and objective data related to frailty were incorporated, key risk factors identified, and frailty prediction models for community-dwelling older adults were developed using multiple ML algorithms. The importance of each variable was also visualized, and a web-based calculator was developed. The aim of our research is to identify high-risk elderly individuals in the community who are susceptible to frailty, thereby enabling healthcare professionals to accurately assess the risk and associated factors of frailty among community-dwelling older adults. This provides a basis for the formulation of appropriate intervention plans, contributing to the delay in the progression of frailty in clinical practice.

## Methods

**Figure 1.**
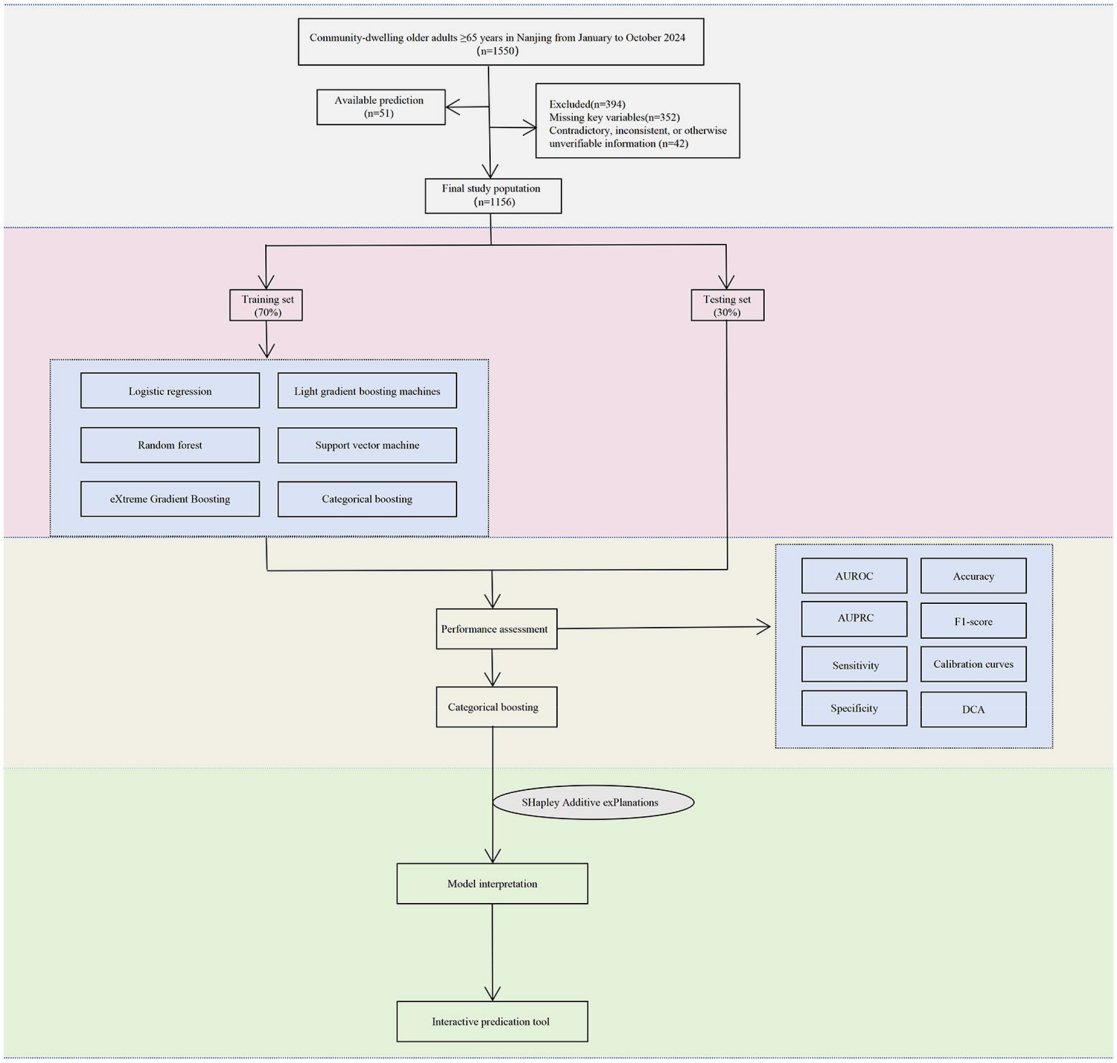
Schematic of the study workflow. AUROC, area under the receiver operating characteristic curve; AUPRC, area under precision-recall curve; DCA, decision curve analysis.

The study design was conducted in accordance with the Transparent Reporting of a multivariable prediction model for Individual Prognosis Or Diagnosis (TRIPOD) and TRIPOD+AI[21] (Supplementary Table S1). The overall workflow is illustrated in Figure 1.

### Design and participants

A retrospective cross-sectional study was conducted to evaluate the health status of community-dwelling older adults in Nanjing. Data covering the period from January to October 2024 were retrieved from an integrated database of electronic health records and historical questionnaire surveys. A multistage stratified random sampling strategy was employed. Initially, 31 community health centers were randomly selected across all 12 administrative districts of Nanjing to ensure geographic representation. Subsequently, 50 individuals aged ≥ 65 years were randomly recruited from each center for the final analysis. The inclusion criteria were as follows: (1) Underwent a health examination at one of the 31 sampled community health centers in Nanjing between January and October 2024; (2) Were aged 65 years or older at the time of examination; (3) Had complete data available in the integrated database, which includes electronic health records and historical questionnaire responses. The exclusion criteria were as follows: (1) Missing key variables (e.g. age, gender, or core health indicators) required for the analysis; (2) Contradictory, inconsistent, or otherwise unverifiable information in the historical questionnaire data.

### Frailty status assessment

Frailty was assessed using the Clinical Frailty Scale (CFS). This widely validated global tool is particularly suitable for large-scale community screening because it efficiently integrates information on physical function, cognition, and comorbidities [22,23]. Studies have demonstrated that CFS scores correlate strongly with important clinical outcomes such as mortality, highlighting its clear prognostic relevance in frailty assessment [24]. In this study, the CFS was employed with scores ranging from 1 (very fit) to 9 (terminally ill). Consistent with the criteria established by Rockwood et al [25], a CFS score of ≥5 (representing “Mildly Frail”) was used as the threshold for clinical frailty. This cutoff marks a distinct transition in health status and is associated with a significantly increased risk of adverse outcomes. Scores of ≤4 indicate a non-frail state, while scores of ≥5 indicate a frail state. The CFS includes the Activities of Daily Living (ADL) scale (feeding, bathing, grooming, dressing, bowel control, bladder control, toilet use, transfers (bed to chair and back), mobility on level surfaces, stairs) and the Instrumental Activities of Daily Living (IADL) scale (shopping, taking transportation, meal preparation, household organization, laundry management, telephone usage, medication adherence, and financial management).

### Data collection

(1) Demographic Characteristics: sociodemographic characteristics include age, gender, marital status, educational level, accompany situation, economic status, and BMI; (2) Lifestyle Characteristics: regarding lifestyle behaviors, the questionnaire surveyed the older adults’ smoking situation and drinking situation. Dietary habits, including meals regular, the daily intake of sugar, salt, and oil, as well as the types and amounts of fresh vegetables and fruits consumed each day; (3) Disease Characteristics: medical history data of diagnosed chronic diseases (hypertension, diabetes mellitus, hyperlipidemia, fatty liver, coronary heart disease, renal insufficiency, osteoporosis, cerebral infarction, cancer) were recorded and consultation frequency. Participants who have taken five or more medications are considered to have polypharmacy; (4) Cognitive Status: cognitive status of the older adults was evaluated using the Mini-Mental State Examination (MMSE). Scores of 27–30 indicate normal cognition; 21–26 indicate mild cognitive impairment; 10–20 indicate moderate cognitive impairment; and 0–9 indicate severe cognitive impairment; (5) Sleep Status: the Pittsburgh Sleep Quality Index (PSQI) was used to assess the sleep quality of the older adults. Scores of 0–5 indicate very good sleep quality; 6–10 indicate fair sleep quality; 11–15 indicate average sleep quality; and 16–21 indicate very poor sleep quality; (6) biomarkers: white blood cell count, neutrophil percentage, neutrophil count, red blood cell count, hemoglobin concentration, platelet count, triglycerides, total cholesterol, low-density lipoprotein, high-density lipoprotein, total bilirubin, alanine aminotransferase, aspartate aminotransferase, creatinine, fasting blood glucose; Neutrophil-to-lymphocyte ratio; Platelet-to-lymphocyte ratio; Systemic immune-inflammation index.

### Sample size calculation

The sample size of the binary outcome prediction model used in this study was calculated as follows [26]: $\text{N }=\text{ exp}\left(\frac{-\text{0.508 + 0.259 ln (}\varphi \text{)+0.504 ln (P)}-\text{ ln (MAPE)}}{\text{0.544}}\right)$. In this formula, φ is the proportion of ending events (φ ****= 0.22), P is the number of predictors (*p* = 9), MAPE (mean absolute percentage error) is the average absolute error between the observed and true outcome probability (MAPE = 0.05). According to calculation, the minimum required sample size for the training set is 690. Therefore, the total dataset (*n* = 1,156) was randomly split into a training set (*n* = 816) and a testing set (*n* = 340) at a 7:3 ratio, which meets the required sample size criteria.

## Data analysis

Prior to data analysis, candidate predictors exhibiting more than 20% missing data were excluded from the dataset. For variables with missing data less than 20%, missing values for continuous variables were imputed using the k-nearest neighbors (KNN) method, while the mode imputation method was applied to categorical variables [27]. Continuous variables were standardized through Z-score normalization, and categorical variables were transformed *via* one-hot encoding [28,29]. The Shapiro-Wilk test was employed to assess the normality of continuous variables. For continuous variables that followed a normal distribution, data were expressed as mean ± standard deviation (SD) and compared using the independent samples t-test. Continuous variables that were not normally distributed were expressed as the median and interquartile range (IQR) and compared using the Mann-Whitney U test. Categorical variables were summarized as frequencies and percentages, with comparisons conducted using the Chi-squared or Fisher’s exact test. The DeLong test was utilized to compare different models based on the Area Under the Receiver Operating Characteristic Curve (AUROC) [30]. A p-value of less than 0.05 (two- tailed) was considered statistically significant. All statistical analyses were performed using R software (version 4.3.3).

### Model development

Participants were randomly divided into training and testing datasets using a 7:3 ratio to facilitate model development and validation, respectively. Initially, univariate analyses were conducted to identify variables that were significantly associated with frailty (*p* < 0.05). Following this, multivariate analysis was performed utilizing the Least Absolute Shrinkage and Selection Operator (LASSO) algorithm to select variables with non-zero coefficients for subsequent investigation [31]. To assess multicollinearity among the selected variables, the variance inflation factor (VIF) was calculated, and any variables exhibiting a VIF greater than 5 were excluded from the final analysis [32].

In this study, six ML models were developed and validated to predict frailty, including logistic regression (LR), extreme gradient boosting (XGBoost), random forest classifier (RF), support vector machine (SVM), light gradient boosting machine (LightGBM), and categorical boosting (CatBoost) models. The optimal hyperparameters were determined using grid search and 10-fold cross-validation, and the best threshold values for the models were identified using the Jordan index. Model validation was performed with a testing set, and performance was evaluated using the AUROC, Area Under the Precision-Recall Curve (AUPRC), accuracy, sensitivity, specificity, and the F1 score. Clinical utility was further assessed *via* decision curve analysis (DCA), which quantifies the net benefit of each model across a range of clinically relevant risk thresholds. Furthermore, calibration performance was assessed using the Brier score, calibration slope, and calibration-in-the-large (CITL). The 95% confidence interval (CI) for AUROC was computed using the DeLong method [30], and the 95% CI for AUPRC was calculated *via* the logit transformation method [33]. The DeLong test was employed for AUROC comparisons. SHapley Additive Explanations (SHAP) were used to visualize the importance of each variable, and a web-based calculator was developed to assist clinicians in daily practice. Statistical analysis was performed using Python software (version 3.11.7).

## Results

### Sample characteristics

A total of 1,156 participants were enrolled in this study, of whom 258 experienced frailty, resulting in an incidence rate of 22.3%. The dataset (*n* = 1,156) was randomly divided into a training set (*n* = 816) and a testing set (*n* = 340) in a 7:3 ratio. The training set participants’ characteristics were summarized in Table 1, and Supplementary Table S2 presents the characteristics of the full dataset along with the missing rate for each variable.

**Table 1.** Demographics and potential risk factors of patients in the training set.

Variables	Overall (*n* = 816)	Non-Frailty (*n* = 620)	Frailty (*n* = 196)	P
Male	381 (46.7)	301 (48.5)	80 (40.8)	0.07
Marital				0.054
Unmarried	7 (0.9)	6 (1.0)	1 (0.5)	
Married	695 (85.2)	538 (86.8)	157 (80.1)	
Divorced	10 (1.2)	8 (1.3)	2 (1.0)	
Widowed	104 (12.7)	68 (11.0)	36 (18.4)	
Education				0.023
Below junior high school	647 (79.3)	478 (77.1)	169 (86.2)	
Senior high school	112 (13.7)	94 (15.2)	18 (9.2)	
College or above	57 (7.0)	48 (7.7)	9 (4.6)	
Accompany				0.062
No	100 (12.3)	68 (11.0)	32 (16.3)	
Yes	716 (87.7)	552 (89.0)	164 (83.7)	
Economics, ¥				0.954
<3000	387 (47.4)	294 (47.4)	93 (47.4)	
3000–5000	334 (40.9)	254 (41.0)	80 (40.8)	
5000-8000	70 (8.6)	52 (8.4)	18 (9.2)	
>8000	25 (3.1)	20 (3.2)	5 (2.6)	
Hypertension				0.345
No	249 (30.5)	195 (31.5)	54 (27.6)	
Yes	567 (69.5)	425 (68.5)	142 (72.4)	
Diabetes Mellitus				0.19
No	513 (62.9)	398 (64.2)	115 (58.7)	
Yes	303 (37.1)	222 (35.8)	81 (41.3)	
Hyperlipidemia				1
No	714 (87.5)	542 (87.4)	172 (87.8)	
Yes	102 (12.5)	78 (12.6)	24 (12.2)	
Fatty Liver				0.438
No	758 (92.9)	573 (92.4)	185 (94.4)	
Yes	58 (7.1)	47 (7.6)	11 (5.6)	
Coronary Heart Disease				0.375
No	755 (92.5)	577 (93.1)	178 (90.8)	
Yes	61 (7.5)	43 (6.9)	18 (9.2)	
Renal Insufficiency				0.204
No	795 (97.4)	607 (97.9)	188 (95.9)	
Yes	21 (2.6)	13 (2.1)	21 (2.6)	
Osteoporosis				0.811
No	799 (97.9)	608 (98.1)	191 (97.4)	
Yes	17 (2.1)	12 (1.9)	5 (2.6)	
Cerebral Infarction				0.003
No	697 (85.4)	543 (87.6)	154 (78.6)	
Yes	119 (14.6)	77 (12.4)	42 (21.4)	
Cancer				0.262
No	794 (97.3)	606 (97.7)	188 (95.9)	
Yes	22 (2.7)	14 (2.3)	8 (4.1)	
Consultation Frequency, month/time				0.954
<3	387 (47.4)	294 (47.4)	93 (47.4)	
3-6	334 (40.9)	254 (41.0)	80 (40.8)	
6-12	70 (8.6)	52 (8.4)	18 (9.2)	
>12	25 (3.1)	20 (3.2)	5 (2.6)	
Medicine				0.003
≥5	133 (16.3)	87 (14.0)	46 (23.5)	
<5	683(83.7)	533(86.0)	150(76.5)	
Smoking				0.957
No	690 (84.6)	525 (84.7)	165 (84.2)	
Yes	126 (15.4)	95 (15.3)	31 (15.8)	
Quit Smoking				0.941
No	769 (94.2)	585 (94.4)	184 (93.9)	
Yes	47 (5.8)	35 (5.6)	12 (6.1)	
Drinking				0.345
No	662 (81.1)	508 (81.9)	154 (78.6)	
Yes	154 (18.9)	112 (18.1)	42 (21.4)	
Quit Drinking				0.234
No	791 (96.9)	598 (96.5)	193 (98.5)	
Yes	25 (3.1)	22 (3.5)	3 (1.5)	
Sugar, g/day				0.158
Unclear	12 (1.5)	12 (1.9)	0 (0.0)	
≤25	421 (51.6)	325 (52.4)	96 (49.0)	
25-50	281 (34.4)	209 (33.7)	72 (36.7)	
≥50	102 (12.5)	74 (11.9)	28 (14.3)	
Salt, g/day				0.869
Unclear	19 (2.3)	13 (2.1)	6 (3.1)	
≤6	494 (60.5)	378 (61.0)	116 (59.2)	
6–10	235 (28.8)	178 (28.7)	57 (29.1)	
≥10	68 (8.3)	51 (8.2)	17 (8.7)	
Oil, g/day				0.447
Unclear	22 (2.7)	19 (3.1)	3 (1.5)	
≤25	444 (54.4)	333 (53.7)	111 (56.6)	
25-50	209 (25.6)	156 (25.2)	53 (27.0)	
≥50	141 (17.3)	112 (18.1)	29 (14.8)	
Meals Regular				0.294
No	7 (0.9)	7 (1.1)	0 (0.0)	
Yes	809 (99.1)	613 (98.9)	196 (100.0)	
Vegetable, g/day				<0.001
<300	181 (22.2)	109 (17.6)	72 (36.7)	
300–500	565 (69.2)	448 (72.3)	117 (59.7)	
>500	70 (8.6)	63 (10.2)	7 (3.6)	
Fruit, g/day				0.15
<200	576 (70.6)	432 (69.7)	144 (73.5)	
200-350	222 (27.2)	171 (27.6)	51 (26.0)	
>350	18 (2.2)	17 (2.7)	1 (0.5)	
Cognitive Status				<0.001
Normal	423 (51.8)	357 (57.6)	66 (33.7)	
Mild	270 (33.1)	207 (33.4)	63 (32.1)	
Moderate	104 (12.7)	52 (8.4)	52 (26.5)	
Severe	19 (2.3)	4 (0.6)	15 (7.7)	
Sleep Quality				0.008
Good	422 (51.7)	338 (54.5)	84 (42.9)	
Fair	279 (34.2)	203 (32.7)	76 (38.8)	
Average	96 (11.8)	63 (10.2)	33 (16.8)	
Poor	19 (2.3)	16 (2.6)	3 (1.5)	
Age, year	72.00 [68.00, 76.00]	71.00 [68.00, 75.00]	75.00 [70.00, 80.00]	<0.001
Body Mass Index, kg/m^2^	24.62 [22.34, 26.73]	24.68 [22.49, 26.81]	24.48 [22.13, 26.28]	0.135
White Blood Cell,10^9^/L	5.94 [5.06, 7.06]	5.96 [5.07, 7.00]	5.91 [4.96, 7.30]	0.843
Neutrophil Percentage,%	60.00 [53.88, 65.50]	59.30 [53.77, 64.90]	62.47 [53.90, 67.05]	0.014
Neutrophil,10^9^/L	3.53 [2.90, 4.40]	3.52 [2.91, 4.29]	3.69 [2.90, 4.68]	0.103
Red Blood Cell,10^12^/L	4.50 [4.20, 4.83]	4.51 [4.21, 4.83]	4.45 [4.16, 4.79]	0.073
Hemoglobin g/L	135.00 [127.00, 143.00]	135.00 [128.00, 144.00]	131.75 [123.75, 140.25]	<0.001
Platelet,10^9^/L	199.45 [168.00, 234.12]	201.00 [170.00, 235.25]	194.50 [161.75, 232.00]	0.109
Triglyceride, mmol/L	1.28 [0.95, 1.82]	1.29 [0.96, 1.83]	1.22 [0.93, 1.70]	0.189
Total Cholesterol, mmol/L	4.60 [3.80, 5.35]	4.66 [3.85, 5.38]	4.50 [3.63, 5.22]	0.031
Low-Density Lipoprotein, mmol/L	2.57 [1.97, 3.21]	2.59 [1.99, 3.20]	2.52 [1.79, 3.21]	0.222
High-Density Lipoprotein, mmol/L	1.35 [1.11, 1.61]	1.35 [1.11, 1.62]	1.30 [1.08, 1.58]	0.359
Total Bilirubin, μmol/L	12.70 [9.97, 16.30]	12.70 [10.00, 16.50]	12.75 [9.65, 15.73]	0.404
Alanine Aminotransferase, u/L	18.00 [13.59, 24.00]	18.48 [14.00, 24.00]	17.00 [13.28, 22.33]	0.091
Aspartate Aminotransferase, u/L	20.90 [17.28, 24.96]	21.00 [17.38, 25.00]	20.59 [17.00, 24.80]	0.529
Creatinine, μmol/L	71.00 [60.80, 83.23]	71.00 [61.66, 83.55]	69.00 [58.75, 82.25]	0.145
Fasting Blood Glucose, mmol/L	5.90 [5.21, 6.95]	5.88 [5.20, 6.89]	6.06 [5.26, 7.30]	0.137
Neutrophil-to-Lymphocyte Ratio	1.92 [1.43, 2.52]	1.87 [1.42, 2.46]	2.09 [1.48, 2.74]	0.006
Platelet-to-Lymphocyte Ratio	108.01 [83.37, 134.21]	108.18 [82.79, 134.14]	107.34 [84.67, 134.58]	0.557
Systemic Immune-Inflammation Index	383.00 [269.67, 529.07]	376.96 [270.48, 517.42]	398.26 [259.68, 559.62]	0.275
Mini-Mental State Examination	27.00 [23.00, 29.00]	27.00 [24.00, 29.00]	24.00 [18.00, 27.25]	<0.001
Pittsburgh Sleep Quality Index	5.00 [3.00, 8.00]	5.00 [3.00, 8.00]	6.00 [4.00, 9.00]	<0.001
Number of Diseases	1.00 [1.00, 2.00]	1.00 [1.00, 2.00]	2.00 [1.00, 2.00]	0.002

Data are presented as n (%), mean (SD), or median (IQR). Variables with normal distribution are presented as mean (standard deviation, SD); Variables without normal distribution are presented as median (interquartile range, IQR).

### Variables selection

To identify variables associated with the risk of frailty in community-dwelling older adults, we analyzed 51 variables. Sequential univariate and LASSO regression analyses were conducted to explore independent risk factors for frailty. In the univariate analysis, age, education, cerebral infarction, medicine, vegetable, cognitive status, sleep quality, number of diseases, neutrophil percentage, hemoglobin, total cholesterol, neutrophil- to-lymphocyte ratio, MMSE, and PSQI were significantly associated with frailty development (*p* < 0.05). The LASSO analysis identified age, education, medicine, vegetable, cognitive status, number of diseases, hemoglobin, total cholesterol, and neutrophil-to-lymphocyte ratio as independent risk factors for frailty. Additionally, the VIF for each variable was less than 5, indicating no multicollinearity among the nine variables (Supplementary Table S3).

### Model performance

Based on the nine independent risk factors for frailty identified by LASSO analysis in the training set, we constructed six prediction models to assess the risk of frailty in community-dwelling older adults. These models were developed using six different ML algorithms: LR, XGBoost, RF, SVM, LightGBM, and CatBoost. The optimal hyperparameter combinations for each model are provided in Supplementary Table S4.

**Figure 2.**
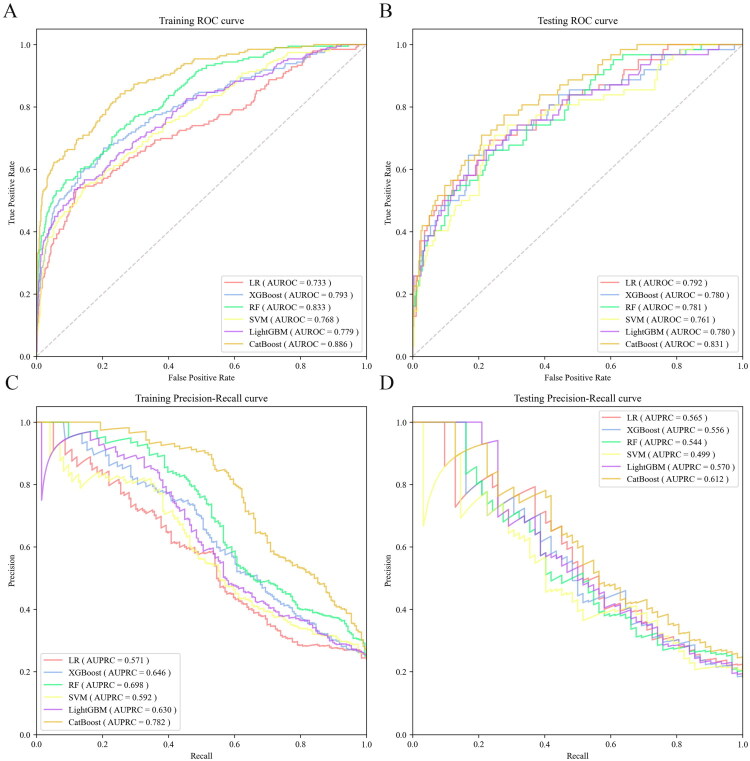
AUROC and AUPRC curves of the six machine learning models. (A) AUROC curves of the training set (B) AUROC curves of the testing set (C) AUPRC curves of the training set (D) AUPRC curves of the testing set. Abbreviations: LR, logistic regression; XGBoost, extreme gradient boosting; RF, random forest; SVM, support vector machine; LightGBM, light gradient boosting machines; CatBoost, categorical boosting; AUROC, area under the receiver operating characteristic curve; AUPRC, area under precision-recall curve.

The predictive performance of these six ML models was evaluated in the training set using the AUROC. The CatBoost (AUROC = 0.886) performed best in predicting the risk of frailty in this population, followed by RF (AUROC = 0.833), the XGBoost (AUROC = 0.793), LightGBM (AUROC = 0.779), SVM (AUROC = 0.768), and LR (AUROC = 0.733) (Figure 2A). In the testing set, the CatBoost model again demonstrated the highest AUROC (0.831) (Figure 2B). Additionally, it achieved the highest AUPRC (training: 0.782; testing: 0.612) (Figure 2C&2D) and sensitivity (training: 0.776; testing: 0.774) among all models in both the training and testing sets (Table 2). Furthermore, to address the potential variability associated with a single test-set evaluation, we performed 1000 nonparametric bootstrap samples on the testing set to calculate the mean and 95% confidence intervals of the CatBoost model’s key performance metrics (Supplementary Table S5). The bootstrap results generally supported the stability of the original point estimates for key discriminative metrics. The bootstrapped mean AUROC of 0.831 closely matched the original estimate of 0.831, and its confidence interval ([0.774, 0.883]) remained above 0.5, reinforcing the model’s discriminative capacity. While some metrics exhibited wider confidence intervals (sensitivity: [0.533, 0.903]), the central tendencies across bootstrap samples were largely consistent with the performance observed in the single testing set, indicating that the model’s overall evaluation is reasonably reliable despite expected sampling variability.

**Table 2.** The performance of the six machine learning models under the optimal threshold on the training set and testing set.

Model	subset	AUROC(95 % CI)	AUPRC(95 % CI)	Accuracy	Sensitivity	Specifity	F1-Value
LR	training	0.733(0.689–0.777)	0.571(0.501–0.638)	0.795	0.541	0.876	0.559
	testing	0.792(0.727–0.856)	0.565(0.440–0.682)	0.788	0.581	0.835	0.500
XGBoost	training	0.793(0.754–0.831)	0.646(0.576–0.710)	0.766	0.668	0.797	0.578
	testing	0.780(0.712–0.848)	0.556(0.431–0.674)	0.762	0.645	0.788	0.497
RF	training	0.833(0.800–0.865)	0.698(0.630–0.758)	0.828	0.566	0.911	0.613
	testing	0.781(0.717–0.845)	0.544(0.420–0.663)	0.815	0.516	0.881	0.504
SVM	training	0.768(0.729–0.808)	0.592(0.522–0.658)	0.781	0.551	0.853	0.547
	testing	0.761(0.691–0.832)	0.499(0.378–0.621)	0.747	0.516	0.799	0.427
LightGBM	training	0.779(0.740–0.818)	0.630(0.560–0.695)	0.801	0.536	0.885	0.565
	testing	0.780(0.712–0.848)	0.570(0.445–0.687)	0.806	0.548	0.863	0.507
CatBoost	training	0.886(0.859–0.912)	0.782(0.719–0.835)	0.792	0.776	0.794	0.641
	testing	0.831(0.776–0.885)	0.612(0.486–0.724)	0.724	0.774	0.712	0.505

Abbreviations: LR, logistic regression; XGBoost, extreme gradient boosting; RF, random forest; SVM, support vector machine; LightGBM, light gradient boosting machines; CatBoost, categorical boosting; AUROC, area under the receiver operating characteristic curve; AUPRC, area under precision-recall curve; 95% CI, 95% confidence interval.

The CatBoost model demonstrated good overall calibration, as reflected by a Brier score of 0.121. The Brier score, which ranges from 0 to 1, quantifies the mean squared error between predicted probabilities and observed binary outcomes; lower values indicate better calibration accuracy. The score of 0.121 in this study indicates a small average discrepancy between the predicted frailty risk and observed outcomes, confirming acceptable overall calibration performance. To further evaluate calibration quality, we computed the calibration slope and the CITL. A calibration slope of 1.817 (>1) suggests mild over-discrimination, meaning the model tends to assign slightly higher predicted probabilities to high‑risk individuals and slightly lower probabilities to low‑risk individuals than actually observed. The CITL value was 0.085, which is very close to the ideal value of 0. CITL quantifies the systematic deviation of predicted probabilities from the true risk across the entire study population. This positive value indicates a slight systematic overestimation at the cohort level, meaning that the model’s average predicted risk is marginally higher than the observed frailty incidence. Specifically, the mean predicted risk exceeded the observed event rate by approximately 8.5 percentage points. Although this bias exists, its magnitude is small and generally considered clinically acceptable, especially for a screening model whose primary objective is to identify high-risk individuals. All model’s calibration performance is detailed in Supplementary Table S6 and visualized in Supplementary Figure S1.

DCA was conducted to assess the clinical utility of the predictive models by quantifying their net benefit across various threshold probabilities (Supplementary Figure S2). The CatBoost model exhibited a positive net benefit across a broad threshold spectrum (approximately 0.1–0.8), exceeding the net benefit of both the “None” and “All” reference strategies. Within the clinically pertinent threshold interval of 0.2–0.6, CatBoost maintained a stable net benefit, performing comparably to XGBoost and superiorly to other models. These findings demonstrate the robustness of the CatBoost model in supporting clinical decision-making for frailty risk stratification, thereby facilitating the targeted identification of high-risk individuals and the optimization of intervention plans.

The Delong test confirmed a statistically significant difference in AUROC between the CatBoost model and the other models in the training set and testing set (*p* < 0.05) (Supplementary Table S7). Consequently, the CatBoost model was selected as the final predictive model for this study. The AUROC, AUPRC, accuracy, sensitivity, specificity, and F1 scores for each model are summarized in Table 2.

### Model interpretation and application

**Figure 3.**
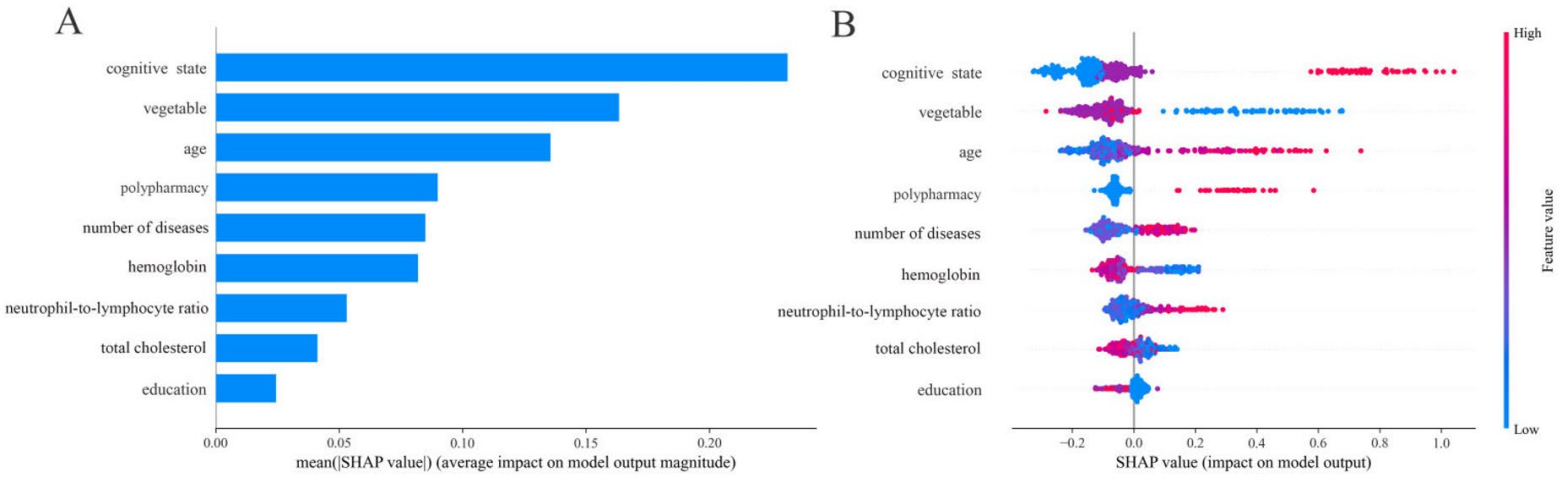
The SHapley Additive exPlanations (SHAP) summary plot for the nine influential variables in the categorical boosting model. (A) The average absolute influence of each factor on the model output magnitude was presented in descending order of feature significance; (B) The graph depicted the dot estimate of the categorical boosting model output, with each dot corresponding to a patient in the dataset.

To enhance the interpretability of the machine learning model, we applied SHAP. Based on the CatBoost model, SHAP analysis was performed to quantify and visually rank the contribution of each candidate predictor to frailty prediction. The results indicated that the most influential predictors, in descending order of importance, were cognitive status, vegetable intake, age, polypharmacy, number of diseases, hemoglobin level, neutrophil-to-lymphocyte ratio, total cholesterol, and education level (Figure 3A).

Further dependency plots illustrated the direction and nature of these associations (Figure 3B). Higher vegetable intake, higher hemoglobin levels, higher total cholesterol, and higher education level were associated with a lower probability of frailty (negative association). Conversely, older age, polypharmacy, greater number of diseases, elevated neutrophil-to-lymphocyte ratio, and poorer cognitive status were associated with an increased risk of frailty (positive association).

**Figure 4.**
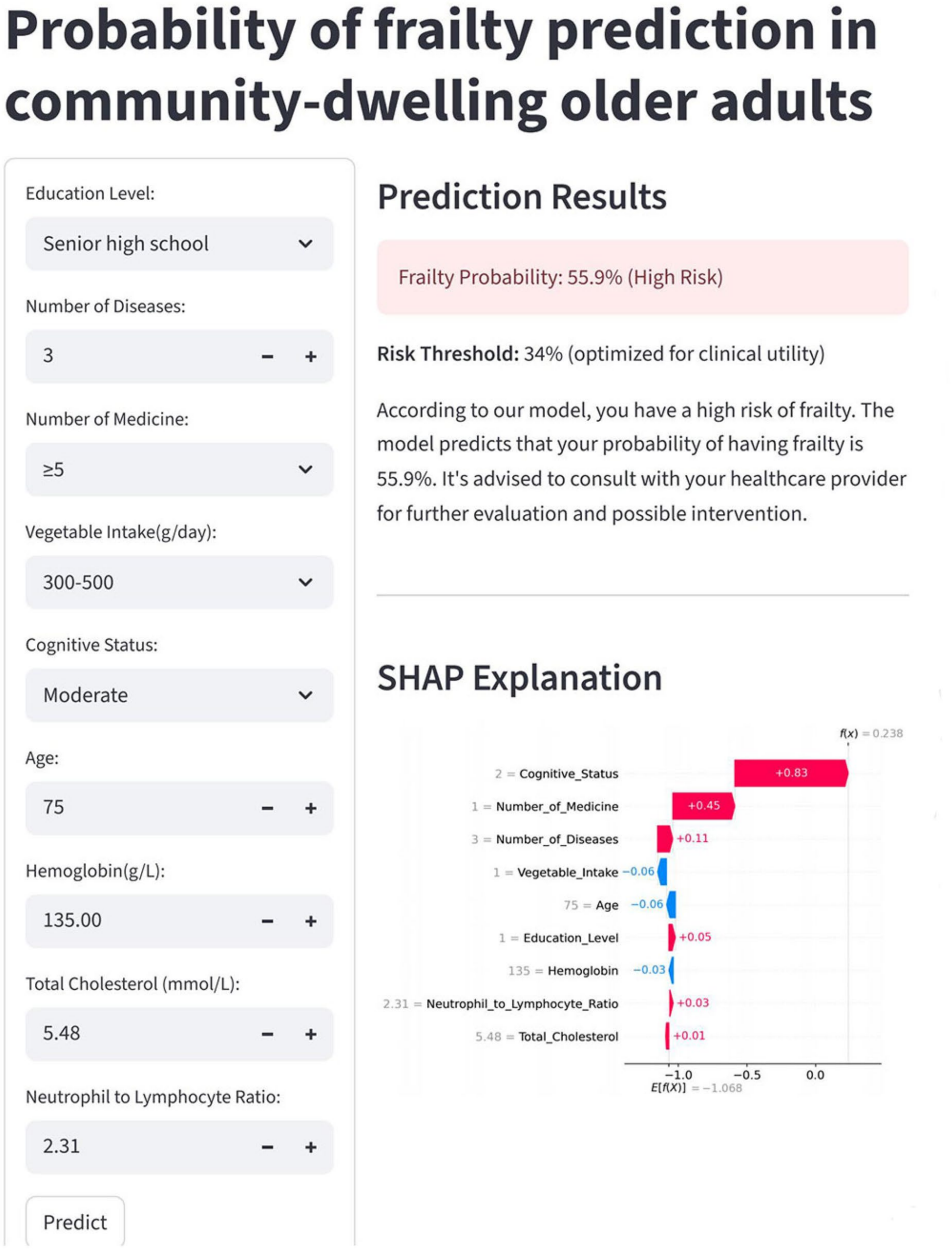
The risk web calculator was designed based on the categorical boosting model.

To enhance the clinical utility of the developed predictive model, we integrated the CatBoost model into a risk network calculator, which is accessible to researchers and clinicians *via* the website (https://community-dwelling-older-adults-frailty-prediction.streamlit.app/). By entering relevant participants information, users can obtain corresponding predictions directly on the webpage (Figure 4).

## Discussion

In this study, the ML prediction model for frailty was initially developed within the older adults of the Nanjing community. Data that were community-specific, readily accessible, and actionable were collected. The analysis identified several significant factors contributing to frailty among community-dwelling older adults, including cognitive status, vegetable consumption, age, HGB levels, NLR, TC, number of diseases, and education. This tool assists general practitioners in assessing and managing patients with chronic diseases. It provides an objective foundation for the early prediction of frailty progression and enables intervention. A key characteristic of this study is the development and internal testing of a risk prediction model tailored for older adults in the Nanjing community. The frailty assessment approach, employing ML algorithms, facilitated the identification of potential influencing factors, thereby enhancing the accuracy of future frailty risk predictions for this population.

The CatBoost model developed in this study shows preliminary potential for frailty screening in this specific community setting. Its satisfactory sensitivity (0.774) is particularly suitable for community screening, facilitating the early identification of individuals at high risk of frailty and reducing the likelihood of missed cases. Although the model shows mild over-discrimination in probability calibration, this does not diminish its core value as a screening tool—namely, the effective identification of high-risk individuals who require further specialized assessment, thereby assisting public health and clinical practitioners in implementing timely, targeted interventions.

Cognitive status was found to be the most strongly associated factor with frailty in our study. Morley et al. [34] proposed that cognitive decline is one of the mechanisms through which frailty develops in older adults. Cognitive impairment and frailty can occur simultaneously, and their interaction accelerates physical decline while diminishing quality of life, creating a detrimental cycle [35]. Age and educational attainment jointly contribute to the prediction of frailty [36,37]. Although age is an immutable risk factor, opportunities may exist to prevent the onset of frailty by proactively enhancing education [38]. Additionally, the presence of multiple comorbidities is common, particularly among older adults who often require long-term treatment involving multiple medications. Overall, the benefits outweigh the harms. However, polypharmacy still inevitably increases the risk of falls, frailty, and death in this population [39,40]. Thus, optimizing medication management for older adults and reducing the use of potentially inappropriate medications may delay the onset of frailty among those with comorbidities.

Notably, the incorporation of biomarkers can enhance both the fairness and accuracy of the model. As individuals age, senescent cells accumulate abnormally and secrete senescence-associated secretory phenotypes [41,42]. This process triggers chronic inflammation, which serves as a key potential mechanism contributing to the development of frailty in older adults. Some studies [43,44] developed prediction models for a community-based population that rely on sociodemographic, self-reported and psychosocial variables, inadequately accounting for objective indicators, such as the NLR, which accurately reflects the state of chronic inflammation. NLR levels increase as an individual becomes more debilitated [45], functioning as a novel chronic inflammatory marker for predicting the onset of frailty events. Diet plays a crucial role in modulating inflammation [46], and diet-induced inflammation may be associated with frailty. Consequently, it is essential to focus on reducing individual dietary inflammation to prevent or delay frailty. This approach can also enhance adherence to and the effectiveness of interventions among older adults.

Except for inflammation, malnutrition significantly contributes to frailty among older adults [47,48]. Biochemical markers such as HGB, TC, and albumin are essential for objective assessment of nutritional status. Older adults with anemia are more likely to experience a debilitated state compared to their healthy counterparts, which may be attributed to anemia’s diminished oxygen-carrying capacity, resulting in tissue hypoxia [49]. Accordingly, issues such as muscle atrophy and reduced aerobic capacity arise. When coupled with the effects of chronic inflammation, HGB levels further decline, exacerbating the severity of frailty. Therefore, implementing interventions such as adequate nutritional supplementation, including increased intake of fruits and vegetables [50], along with enhanced physical activity [51,52], can help improve the anemic status while delaying frailty. Besides, TC levels partially reflect malnutrition and inflammation [53]. Wenying et al. [54] found that lower TC levels were associated with pre-frailty and frailty in both women and men. Appropriate dietary modifications may effectively raise TC levels, thereby delaying the progression of frailty [55].

Finally, we developed an interpretable online web calculator for the first time that enables general practitioners to either use the website directly to identify high-risk people within the community. This aligns with the current trend of accelerated integration between artificial intelligence (AI) and clinical medicine. AI is transforming clinical practice by leveraging machine learning and natural language processing to analyze complex medical data, thereby enhancing diagnostic precision and enabling personalized treatment [56]. Due to the black-box nature of ML models [57], SHAP was utilized to elucidate whether a feature contributes positively or negatively to the model’s predictions. We also visualized this component on a web page, allowing general practitioners to easily and intuitively understand how each feature impacts individuals. As illustrated in Figure 3, the web-based calculator developed through this study indicates a 55.9% frailty risk probability for the patient. SHAP values provide intuitive quantification of risk contributions, revealing cognitive impairment, polypharmacy, and comorbidity burden as the top three influential risk factors. While cognitive status and comorbidity counts are challenging to fully reverse in the short term, substantial evidence demonstrates that multidimensional interventions targeting lifestyle modification, medical optimization, and psychological well-being can significantly delay disease progression, alleviate symptoms, and improve quality of life. The International Working Group on Sarcopenia and Frailty recommends structured deprescribing protocols for older adults with excessive medication regimens. Conversely, vegetable intake emerges as a protective factor, with its negative SHAP value supporting recommendations to maintain or further optimize dietary patterns. In contrast, features such as educational attainment and hemoglobin levels demonstrate relatively low predictive power in this model, suggesting that clinicians may streamline clinical assessments by prioritizing higher-impact variables. This web-based tool not only generates risk scores but also highlights key risk determinants through SHAP visualization, enabling physicians to rapidly identify critical intervention points. Its application model is in line with the grassroots practice of medical AI emphasized by Sadée et al [58]. This study aims to explore a feasible practical path for AI to conduct stratified management of high-risk populations in community settings, and the actual effectiveness of the web-based calculator needs to be further evaluated in future community applications [59–62].

Our study has several limitations. First, cognitive functioning and the degree of frailty were assessed using straightforward scales, acknowledging that the selection of different scales may inherently introduce subjective bias. Second, we adopted mode imputation to handle missing values in categorical variables with very low missing rates (<5%). While this method is simple and widely used, it may underestimate variance and ignore inherent uncertainty. Nevertheless, given the minimal amount of missing data, the associated risk of bias is largely mitigated.Third, The generalizability of our findings is subject to geographic constraints, as the study cohort was recruited exclusively from select community health centers in Nanjing. Consequently, the results primarily reflect the characteristics of an urban, community-dwelling population and may not be fully extrapolatable to other regions—particularly rural areas or populations with distinct socioeconomic, cultural, or healthcare backgrounds. Furthermore, as a monocentric study, a significant limitation is the lack of a real, independent validation cohort. Although the dataset was split into training and testing subsets, this internal validation approach may not fully capture the model’s performance in different clinical settings or diverse geographic populations. Fourth, the online calculator developed in this work serves primarily as a proof‑of‑concept demonstrator and has not yet undergone real-world usability or effectiveness validation in clinical or community environments. Future research should include external validation in diverse, multi-center cohorts and prospective evaluation of the calculator’s usability and performance in practical settings to further assess the robustness, transportability, and translational utility of the model.

## Conclusions

This study explores the feasibility of a machine learning-based tool for screening the risk of frailty among community-dwelling elderly individuals, incorporating key predictive factors such as cognitive status, age, and comorbidities. The tool attempts to integrate community-specific factors and biomarkers with the aim of assisting in the identification of high-risk individuals within similar community contexts. While this tool offers a potential framework for the monitoring and personalized adjustment of prevention and management strategies, its real-world effectiveness and applicability across different regions remain to be established through multicenter studies with independent validation. Future research needs to validate the tool in more diverse community populations and incorporate methodological improvements to comprehensively evaluate its practical application value.

## Supplementary Material

Supplemental Material

## Data Availability

The data sets analyzed during the current study are not publicly available for patient privacy purposes but are available from the corresponding author upon reasonable request.
