# Supplementary material for: An explainable online frailty prediction model for community-dwelling older adults based on machine learning algorithms: a cross-sectional study based on retrospective health data
Source: Ann Med. 2026 Apr 2;58(1):2647569. doi: 10.1080/07853890.2026.2647569 (PMC13047847; doi:10.1080/07853890.2026.2647569)
Supplement: Supplemental Material [file IANN_A_2647569_SM0198.docx]

| **Supplementary Table S1.**TRIPOD+AI checklist. | | | | |
| --- | --- | --- | --- | --- |
| **Section/Topic Item** | | **Development**  **/ evaluation**1 | **Checklist item** | **Reported**  **on page** |
| **TITLE** | | | |  |
| *Title* | 1 | D;E | Identify the study as developing or evaluating the performance of a multivariable prediction model, the target population, and the outcome to be predicted | Page 1 |
| **ABSTRACT** | | | |  |
| *Abstract* | 2 | D;E | See TRIPOD+AI for Abstracts checklist | Page 1 |
| **INTRODUCTION** | | | |  |
| *Background* | 3a | D;E | Explain the healthcare context (including whether diagnostic or prognostic) and rationale for developing or evaluating the prediction model, including references to existing models | Page 2 |
|  | 3b | D;E | Describe the target population and the intended purpose of the prediction model in the context of the care pathway, including its intended users (e.g., healthcare professionals, patients, public) | Page 2 |
|  | 3c | D;E | Describe any known health inequalities between sociodemographic groups | Page 2 |
| *Objectives* | 4 | D;E | Specify the study objectives, including whether the study describes the development or validation of a prediction model (or both) | Page 2 |
| **METHODS** | | | |  |
| *Data* | 5a | D;E | Describe the sources of data separately for the development and evaluation datasets (e.g., randomised trial, cohort, routine care or registry data), the rationale for using these data, and representativeness of the data | Page 3 |
|  | 5b | D;E | Specify the dates of the collected participant data, including start and end of participant accrual; and, if applicable, end of follow-up | Page 3 |
| *Participants* | 6a | D;E | Specify key elements of the study setting (e.g., primary care, secondary care, general population) including the number and location of centres | Page 3 |
|  | 6b | D;E | Describe the eligibility criteria for study participants | Page 3 |
|  | 6c | D;E | Give details of any treatments received, and how they were handled during model development or evaluation, if relevant | Page 3 |
| *Data preparation* | 7 | D;E | Describe any data pre-processing and quality checking, including whether this was similar across relevant sociodemographic groups | Page 3-4 |
| *Outcome* | 8a | D;E | Clearly define the outcome that is being predicted and the time horizon, including how and when  assessed, the rationale for choosing this outcome, and whether the method of outcome assessment is consistent across sociodemographic groups | Page 3 |
|  | 8b | D;E | If outcome assessment requires subjective interpretation, describe the qualifications and demographic characteristics of the outcome assessors | Page 4 |
|  | 8c | D;E | Report any actions to blind assessment of the outcome to be predicted | Page 3 |
| *Predictors* | 9a | D | Describe the choice of initial predictors (e.g., literature, previous models, all available predictors) and any pre-selection of predictors before model building | Page 3 |
|  | 9b | D;E | Clearly define all predictors, including how and when they were measured (and any actions to blind assessment of predictors for the outcome and other predictors) | Page 3 |
|  | 9c | D;E | If predictor measurement requires subjective interpretation, describe the qualifications and demographic characteristics of the predictor assessors | Page 3 |
| *Sample size* | 10 | D;E | Explain how the study size was arrived at (separately for development and evaluation), and justify that the study size was sufficient to answer the research question. Include details of any sample size  calculation | Page 3 |
| *Missing data* | 11 | D;E | Describe how missing data were handled. Provide reasons for omitting any data | Page 3 |
| *Analytical methods* | 12a | D | Describe how the data were used (e.g., for development and evaluation of model performance) in the analysis, including whether the data were partitioned, considering any sample size requirements | Page 3-4 |
|  | 12b | D | Depending on the type of model, describe how predictors were handled in the analyses (functional form, rescaling, transformation, or any standardisation). | Page 3 |
|  | 12c | D | Specify the type of model, rationale2, all model-building steps, including any hyperparameter tuning, and method for internal validation | Page 3-4 |
|  | 12d | D;E | Describe if and how any heterogeneity in estimates of model parameter values and model performance was handled and quantified across clusters (e.g., hospitals, countries). See TRIPOD-Cluster for  additional considerations3 | Page 3 |
|  | 12e | D;E | Specify all measures and plots used (and their rationale) to evaluate model performance (e.g., discrimination, calibration, clinical utility) and, if relevant, to compare multiple models | Page 5 |
|  | 12f | E | Describe any model updating (e.g., recalibration) arising from the model evaluation, either overall or for particular sociodemographic groups or settings | Page 4 |
|  | 12g | E | For model evaluation, describe how the model predictions were calculated (e.g., formula, code, object, application programming interface) | Page 4 |
| *Class imbalance* | 13 | D;E | If class imbalance methods were used, state why and how this was done, and any subsequent methods to recalibrate the model or the model predictions | Page 4 |
| *Fairness* | 14 | D;E | Describe any approaches that were used to address model fairness and their rationale | Page 4 |
| *Model output* | 15 | D | Specify the output of the prediction model (e.g., probabilities, classification). Provide details and rationale for any classification and how the thresholds were identified | Page 4 |

| **Supplementary Table S2.** Demographics and potential risk factors of patients in the dataset. | | | | |  |
| --- | --- | --- | --- | --- | --- |
| Variables | Overall(n=1156) | Non-Frailty(n=898) | Frailty(n=258) | P | Miss% |
| Male | 549 (47.5) | 440 (49.0) | 109 (42.2) | 0.065 |  |
| Marital |  |  |  | 0.01 |  |
| Unmarried | 10 ( 0.9) | 7 ( 0.8) | 3 ( 1.2) |  |  |
| Married | 985 (85.2) | 781 (87.0) | 204 (79.1) |  |  |
| Divorced | 15 ( 1.3) | 12 ( 1.3) | 3 ( 1.2) |  |  |
| Widowed | 146 (12.6) | 98 (10.9) | 48 (18.6) |  |  |
| Education |  |  |  | 0.012 |  |
| Below junior high school | 903 (78.1) | 684 (76.2) | 219 (84.9) |  |  |
| Senior high school | 171 (14.8) | 145 (16.1) | 26 (10.1) |  |  |
| College or above | 82 ( 7.1) | 69 ( 7.7) | 13 ( 5.0) |  |  |
| Accompany |  |  |  | 0.104 |  |
| No | 139 (12.0) | 100 (11.1) | 39 (15.1) |  |  |
| Yes | 1017 (88.0) | 798 (88.9) | 219 (84.9) |  |  |
| Economics, ¥ |  |  |  | 0.921 |  |
| ＜3000 | 547 (47.3) | 422 (47.0) | 125 (48.4) |  |  |
| 3000-5000 | 463 (40.1) | 363 (40.4) | 100 (38.8) |  |  |
| 5000-8000 | 111 ( 9.6) | 87 ( 9.7) | 24 ( 9.3) |  |  |
| ＞8000 | 35 ( 3.0) | 26 ( 2.9) | 9 ( 3.5) |  |  |
| Hypertension |  |  |  | 0.131 |  |
| No | 351 (30.4) | 283 (31.5) | 68 (26.4) |  |  |
| Yes | 805 (69.6) | 615 (68.5) | 190 (73.6) |  |  |
| Diabetes Mellitus |  |  |  | 0.376 |  |
| No | 746 (64.5) | 586 (65.3) | 160 (62.0) |  |  |
| Yes | 410 (35.5) | 312 (34.7) | 98 (38.0) |  |  |
| Hyperlipidemia |  |  |  | 0.974 |  |
| No | 1020 (88.2) | 793 (88.3) | 227 (88.0) |  |  |
| Yes | 136 (11.8) | 105 (11.7) | 31 (12.0) |  |  |
| Fatty Liver |  |  |  | 0.633 |  |
| No | 1079 (93.3) | 836 (93.1) | 243 (94.2) |  |  |
| Yes | 77 ( 6.7) | 62 ( 6.9) | 15 ( 5.8) |  |  |
| Coronary Heart Disease |  |  |  | 0.173 |  |
| No | 1061 (91.8) | 830 (92.4) | 231 (89.5) |  |  |
| Yes | 95 ( 8.2) | 68 ( 7.6) | 27 (10.5) |  |  |
| Renal Insufficiency |  |  |  | 0.119 |  |
| No | 1132 (97.9) | 883 (98.3) | 249 (96.5) |  |  |
| Yes | 24 ( 2.1) | 15 ( 1.7) | 9 ( 3.5) |  |  |
| Osteoporosis |  |  |  | 0.063 |  |
| No | 1134 (98.1) | 885 (98.6) | 249 (96.5) |  |  |
| Yes | 22 ( 1.9) | 13 ( 1.4) | 9 ( 3.5) |  |  |
| Cerebral Infarction |  |  |  | 0.001 |  |
| No | 994 (86.0) | 789 (87.9) | 205 (79.5) |  |  |
| Yes | 162 (14.0) | 109 (12.1) | 53 (20.5) |  |  |
| Cancer |  |  |  | 0.419 |  |
| No | 1130 (97.8) | 880 (98.0) | 250 (96.9) |  |  |
| Yes | 26 ( 2.2) | 18 ( 2.0) | 8 ( 3.1) |  |  |
| Consultation Frequency, month/time |  |  |  | 0.456 | 4.6% |
| ＜3 | 665 (57.5) | 515 (57.3) | 150 (58.1) |  |  |
| 3-6 | 201 (17.4) | 150 (16.7) | 51 (19.8) |  |  |
| 6-12 | 168 (14.5) | 133 (14.8) | 35 (13.6) |  |  |
| ＞12 | 122 (10.6) | 100 (11.1) | 22 ( 8.5) |  |  |
| Medicine |  |  |  | <0.001 |  |
| ≥5 | 169 (14.6) | 111 (12.4) | 58 (22.5) |  |  |
| ＜5 | 987(85.4) | 787(79.7) | 200(20.3) |  |  |
| Smoking |  |  |  | 0.417 |  |
| No | 971 (84.0) | 759 (84.5) | 212 (82.2) |  |  |
| Yes | 185 (16.0) | 139 (15.5) | 46 (17.8) |  |  |
| Quit Smoking |  |  |  | 1 | 3.4% |
| No | 1083 (93.7) | 841 (93.7) | 242 (93.8) |  |  |
| Yes | 73 ( 6.3) | 57 ( 6.3) | 16 ( 6.2) |  |  |
| Drinking |  |  |  | 0.833 | 0.5% |
| No | 935 (80.9) | 728 (81.1) | 207 (80.2) |  |  |
| Yes | 221 (19.1) | 170 (18.9) | 51 (19.8) |  |  |
| Quit Drinking |  |  |  | 0.303 | 4.4% |
| No | 1120 (96.9) | 867 (96.5) | 253 (98.1) |  |  |
| Yes | 36 ( 3.1) | 31 ( 3.5) | 5 ( 1.9) |  |  |
| Sugar, g/day |  |  |  | 0.045 |  |
| Unclear | 20 ( 1.7) | 20 ( 2.2) | 0 ( 0.0) |  |  |
| ≤25 | 587 (50.8) | 464 (51.7) | 123 (47.7) |  |  |
| 25-50 | 396 (34.3) | 298 (33.2) | 98 (38.0) |  |  |
| ≥50 | 153 (13.2) | 116 (12.9) | 37 (14.3) |  |  |
| Oil, g/day |  |  |  | 0.898 |  |
| Unclear | 25 ( 2.2) | 18 ( 2.0) | 7 ( 2.7) |  |  |
| ≤6 | 706 (61.1) | 551 (61.4) | 155 (60.1) |  |  |
| 6-10 | 327 (28.3) | 254 (28.3) | 73 (28.3) |  |  |
| ≥10 | 98 ( 8.5) | 75 ( 8.4) | 23 ( 8.9) |  |  |
| Salt, g/day |  |  |  | 0.444 |  |
| Unclear | 34 ( 2.9) | 30 ( 3.3) | 4 ( 1.6) |  |  |
| ≤25 | 630 (54.5) | 490 (54.6) | 140 (54.3) |  |  |
| 25-50 | 296 (25.6) | 225 (25.1) | 71 (27.5) |  |  |
| ≥50 | 196 (17.0) | 153 (17.0) | 43 (16.7) |  |  |
| Meals Regular |  |  |  | 0.683 | 3.9% |
| No | 9 ( 0.8) | 8 ( 0.9) | 1 ( 0.4) |  |  |
| Yes | 1147 (99.2) | 890 (99.1) | 257 (99.6) |  |  |
| Vegetable, g/day |  |  |  | <0.001 | 3.6% |
| ＜300 | 259 (22.4) | 157 (17.5) | 102 (39.5) |  |  |
| 300-500 | 800 (69.2) | 656 (73.1) | 144 (55.8) |  |  |
| ＞500 | 97 ( 8.4) | 85 ( 9.5) | 12 ( 4.7) |  |  |
| Fruit, g/day |  |  |  | 0.06 | 4.8% |
| ＜200 | 814 (70.4) | 620 (69.0) | 194 (75.2) |  |  |
| 200-350 | 316 (27.3) | 254 (28.3) | 62 (24.0) |  |  |
| ＞350 | 26 ( 2.2) | 24 ( 2.7) | 2 ( 0.8) |  |  |
| Cognitive Status |  |  |  | <0.001 |  |
| Normal | 600 (51.9) | 518 (57.7) | 82 (31.8) |  |  |
| Mild | 378 (32.7) | 296 (33.0) | 82 (31.8) |  |  |
| Moderate | 154 (13.3) | 80 ( 8.9) | 74 (28.7) |  |  |
| Severe | 24 ( 2.1) | 4 ( 0.4) | 20 ( 7.8) |  |  |
| Sleep Quality |  |  |  | <0.001 |  |
| Good | 592 (51.2) | 487 (54.2) | 105 (40.7) |  |  |
| Fair | 399 (34.5) | 301 (33.5) | 98 (38.0) |  |  |
| Average | 139 (12.0) | 91 (10.1) | 48 (18.6) |  |  |
| Poor | 26 ( 2.2) | 19 ( 2.1) | 7 ( 2.7) |  |  |
| Age, year | 72.00 [68.00, 76.00] | 71.00 [68.00, 75.00] | 75.00 [71.00, 80.00] | <0.001 |  |
| Body Mass Index, kg/m^2^ | 24.68 [22.38, 26.81] | 24.78 [22.51, 26.91] | 24.22 [22.16, 26.26] | 0.043 |  |
| White Blood Cell,10^9^/L | 5.98 [5.10, 7.08] | 6.00 [5.13, 7.04] | 5.94 [5.05, 7.22] | 0.958 | 0.5% |
| Neutrophil Percentage | 59.80 [54.08, 65.31] | 59.50 [54.20, 64.90] | 60.90 [53.80, 66.69] | 0.066 | 17.9% |
| Neutrophil,10^9^/L | 3.56 [2.93, 4.41] | 3.54 [2.97, 4.36] | 3.68 [2.86, 4.55] | 0.545 | 18.2% |
| Red Blood Cell,10^12^/L | 4.50 [4.20, 4.84] | 4.52 [4.22, 4.85] | 4.44 [4.11, 4.79] | 0.003 | 15.4% |
| Hemoglobin g/L | 135.00 [127.00, 144.00] | 136.00 [128.00, 144.50] | 132.00 [125.00, 141.00] | <0.001 | 13.2% |
| Platelet,10^9^/L | 199.00 [165.00, 235.00] | 200.00 [167.00, 236.00] | 195.00 [161.25, 232.75] | 0.2 | 4.1% |
| Triglyceride, mmol/L | 1.31 [0.95, 1.87] | 1.32 [0.96, 1.90] | 1.24 [0.93, 1.70] | 0.062 | 0.6% |
| Total Cholesterol, mmol/L | 4.60 [3.85, 5.39] | 4.66 [3.91, 5.40] | 4.52 [3.69, 5.26] | 0.051 | 0.8% |
| Low-Density Lipoprotein, mmol/L | 2.60 [1.99, 3.24] | 2.63 [2.01, 3.24] | 2.54 [1.88, 3.23] | 0.237 | 0.7% |
| High-Density Lipoprotein, mmol/L | 1.35 [1.10, 1.61] | 1.35 [1.11, 1.62] | 1.31 [1.09, 1.59] | 0.53 | 0.9% |
| Total Bilirubin, μmol/L | 12.63 [10.00, 16.20] | 12.66 [10.00, 16.20] | 12.60 [9.71, 15.58] | 0.366 | 1.2% |
| Alanine Aminotransferase, u/L | 18.00 [14.00, 24.00] | 18.42 [14.00, 24.00] | 17.00 [13.40, 22.62] | 0.082 | 0.6% |
| Aspartate Aminotransferase, u/L | 21.00 [17.65, 25.00] | 21.00 [17.70, 25.00] | 20.69 [17.15, 25.00] | 0.27 | 0.8% |
| Creatinine, μmol/L | 70.90 [60.77, 83.55] | 71.00 [61.00, 83.50] | 70.12 [60.00, 83.76] | 0.479 | 1% |
| Fasting Blood Glucose, mmol/L | 5.91 [5.22, 6.92] | 5.90 [5.22, 6.90] | 5.98 [5.25, 7.03] | 0.551 | 1.3% |
| Neutrophil-to-Lymphocyte Ratio | 1.91 [1.44, 2.49] | 1.88 [1.43, 2.46] | 2.04 [1.44, 2.71] | 0.036 |  |
| Platelet-to-Lymphocyte Ratio | 106.88 [82.33, 134.31] | 106.62 [82.01, 134.43] | 107.14 [83.47, 134.08] | 0.483 |  |
| Systemic Immune-Inflammation Index | 378.32 [270.40, 523.67] | 377.24 [273.64, 521.29] | 383.86 [260.34, 534.27] | 0.568 |  |
| Mini-Mental State Examination | 27.00 [23.00, 29.00] | 27.00 [24.00, 29.00] | 23.50 [17.00, 27.00] | <0.001 |  |
| Pittsburgh Sleep Quality Index | 5.00 [3.00, 8.00] | 5.00 [3.00, 8.00] | 6.00 [4.00, 10.00] | <0.001 |  |
| Number of Diseases | 1.00 [1.00, 2.00] | 1.00 [1.00, 2.00] | 2.00 [1.00, 2.00] | <0.001 |  |
| Data are presented as n (%), mean (SD), or median (IQR); Variables with normal distribution are presented as mean (standard deviation, SD); Variables without normal distribution are presented as median (interquartile range, IQR); Miss%, missing percentage.; Variables without a “Miss%” value have complete data. | | | | | |

| **Supplementary Table S3.** Collinearity analysis of related variables. | |
| --- | --- |
| **Variables** | **Variation inflation factors** |
| Education | 1.06 |
| Medicine | 1.27 |
| Vegetable | 1.03 |
| Cognitive state | 1.23 |
| Age | 1.23 |
| Hemoglobin | 1.07 |
| Total cholesterol | 1.06 |
| Neutrophil-to-Lymphocyte ratio | 1.05 |
| Number of diseases | 1.26 |

| **Supplementary Table S4.** The optimal hyperparameters of the six machine learning models. | | |
| --- | --- | --- |
| **Model** | **Hyperparameter** | **Optimal value** |
| LR | c | 0.1 |
|  | penalty | l2 |
|  | solver | liblinear |
|  | class weight | balanced |
|  | random state | 42 |
| XGBoost | colsample bytree | 1 |
|  | gamma | 0.5 |
|  | learning rate | 0.1 |
|  | max depth | 4 |
|  | n estimators | 13 |
|  | min child weight | 4 |
|  | subsample | 0.5 |
|  | max leaves | 31 |
|  | class weight | balanced |
|  | random state | 42 |
| RF | n estimators | 100 |
|  | max depth | 4 |
|  | min samples leaf | 2 |
|  | min samples split | 2 |
|  | max features | sqrt |
|  | class weight | balanced |
|  | random state | 42 |
| SVM | kernel | poly |
|  | c | 1 |
|  | gamma | 0.05 |
|  | decision function shape | ovo |
|  | verbose | 3 |
|  | probability | True |
|  | class weight | balanced |
|  | random state | 42 |
| LightGBM | bagging fraction | 0.8 |
|  | feature fraction | 0.6 |
|  | learning rate | 0.02 |
|  | max depth | 2 |
|  | n estimators | 200 |
|  | min data in leaf | 1 |
|  | num leaves | 3 |
|  | class weight | balanced |
|  | random state | 42 |
| CatBoost | iterations | 50 |
|  | max depth | 12 |
|  | learning rate | 0.07 |
|  | metric period | 50 |
|  | subsample | 0.9 |
|  | random state | 42 |
| **Abbreviations:** LR, logistic regression; XGBoost, extreme gradient boosting; RF, random forest; SVM, support vector machine; LightGBM, light gradient boosting machines; CatBoost, categorical boosting. | | |

| **Supplementary Table S5**. Bootstrapped performance metrics of the CatBoost model in the testing set. | |
| --- | --- |
| **Metrics** | **Bootstrap Mean (95% CI)** |
| AUROC | 0.831(0.774,0.883) |
| AUPRC | 0.617(0.494,0.722) |
| Accuracy | 0.772(0.662.0.880) |
| Sensitivity | 0.746(0.533,0.903) |
| Specificity | 0.778(0.617,0.949) |
| F1 | 0.546(0.442,0.654) |
| **Abbreviations**: CatBoost, categorical boosting; 95% CI, 95% confidence interval; AUROC, area under the receiver operating characteristic curve; AUPRC, area under precision-recall curve. | |

| **Supplementary Table 6**. Calibration performance metrics of the six machine learning models. | | | |
| --- | --- | --- | --- |
| **Model** | **Brier Score** | **CITL** | **Calibration Slope** |
| LR | 0.196 | 0.272 | 1.06 |
| XGBoost | 0.124 | 0.061 | 1.907 |
| RF | 0.181 | 0.246 | 1.512 |
| SVM | 0.126 | 0.064 | 1.03 |
| LightGBM | 0.193 | 0.272 | 1.723 |
| CatBoost | 0.121 | 0.085 | 1.817 |

| **Supplementary Table S7.** Statistical difference of the AUROC of six machine learning models. (Delong test) | | | | | | | |
| --- | --- | --- | --- | --- | --- | --- | --- |
|  | Model | LR | XGBoost | RF | SVM | LightGBM | CatBoost |
| Training set | LR | \ | <0.001 | <0.001 | <0.001 | <0.001 | <0.001 |
|  | XGBoost | <0.001 | \ | <0.001 | 0.0373 | 0.1668 | <0.001 |
|  | RF | <0.001 | <0.001 | \ | <0.001 | <0.001 | <0.001 |
|  | SVM | <0.001 | 0.0373 | <0.001 | \ | 0.2963 | <0.001 |
|  | LightGBM | <0.001 | 0.1668 | <0.001 | 0.2963 | \ | <0.001 |
|  | CatBoost | <0.001 | <0.001 | <0.001 | <0.001 | <0.001 | \ |
| Testing set | LR | \ | 0.5801 | 0.5252 | 0.1080 | 0.4239 | 0.0369 |
|  | XGBoost | 0.5801 | \ | 0.9643 | 0.5296 | 0.9882 | 0.0173 |
|  | RF | 0.5252 | 0.9643 | \ | 0.4308 | 0.9237 | 0.0056 |
|  | SVM | 0.1080 | 0.5296 | 0.4308 | \ | 0.4613 | 0.0034 |
|  | LightGBM | 0.4239 | 0.9882 | 0.9237 | 0.4613 | \ | 0.0140 |
|  | CatBoost | 0.0369 | 0.0173 | 0.0056 | 0.0034 | 0.0140 | \ |
| **Abbreviations:** LR, logistic regression; XGBoost, extreme gradient boosting; RF, random forest; SVM, support vector machine; LightGBM, light gradient boosting machines; CatBoost, categorical boosting; AUROC, area under the receiver operating characteristic curve. | | | | | | | |


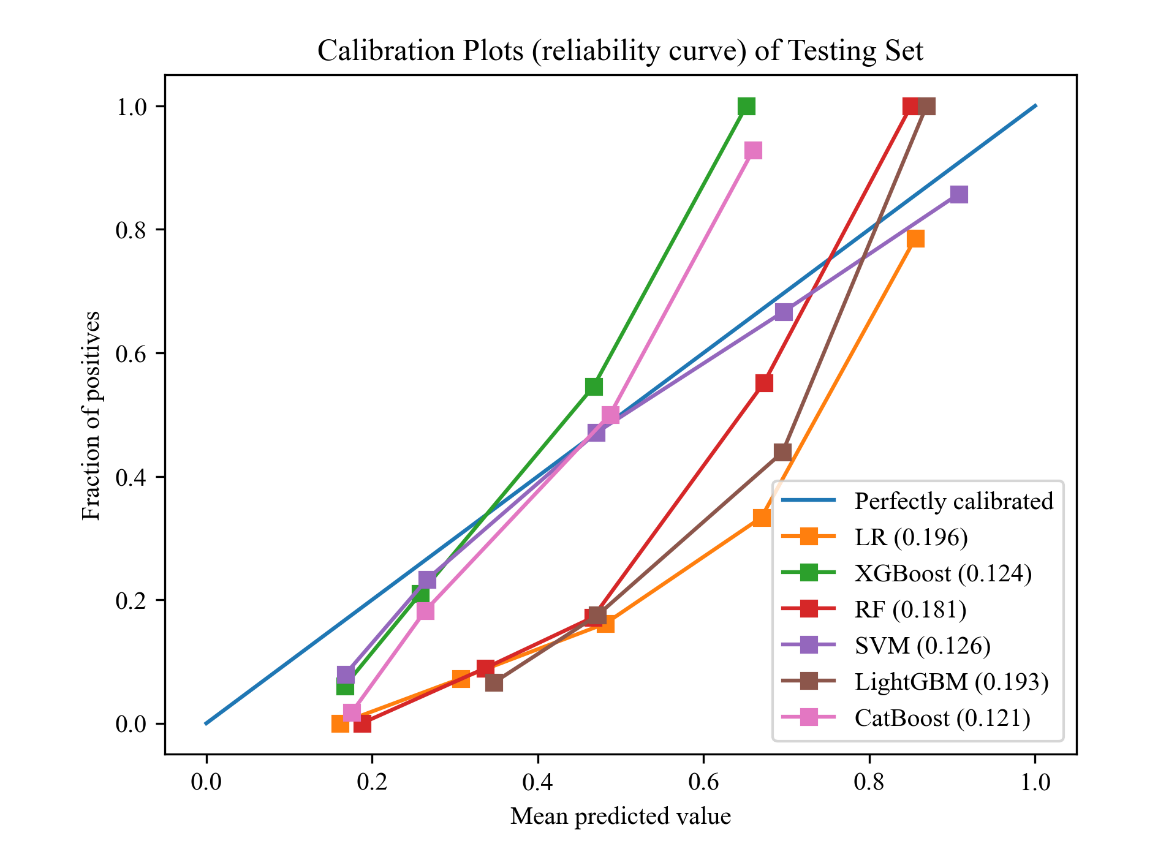


**Supplementary Figure S1.** Calibration plots for the probability of frailty prediction for community-dwelling older adults from the six machine learning models in the testing set. **Abbreviations:** LR, logistic regression; XGBoost, extreme gradient boosting; RF, random forest; SVM, support vector machine; LightGBM, light gradient boosting machines; CatBoost, categorical boosting.


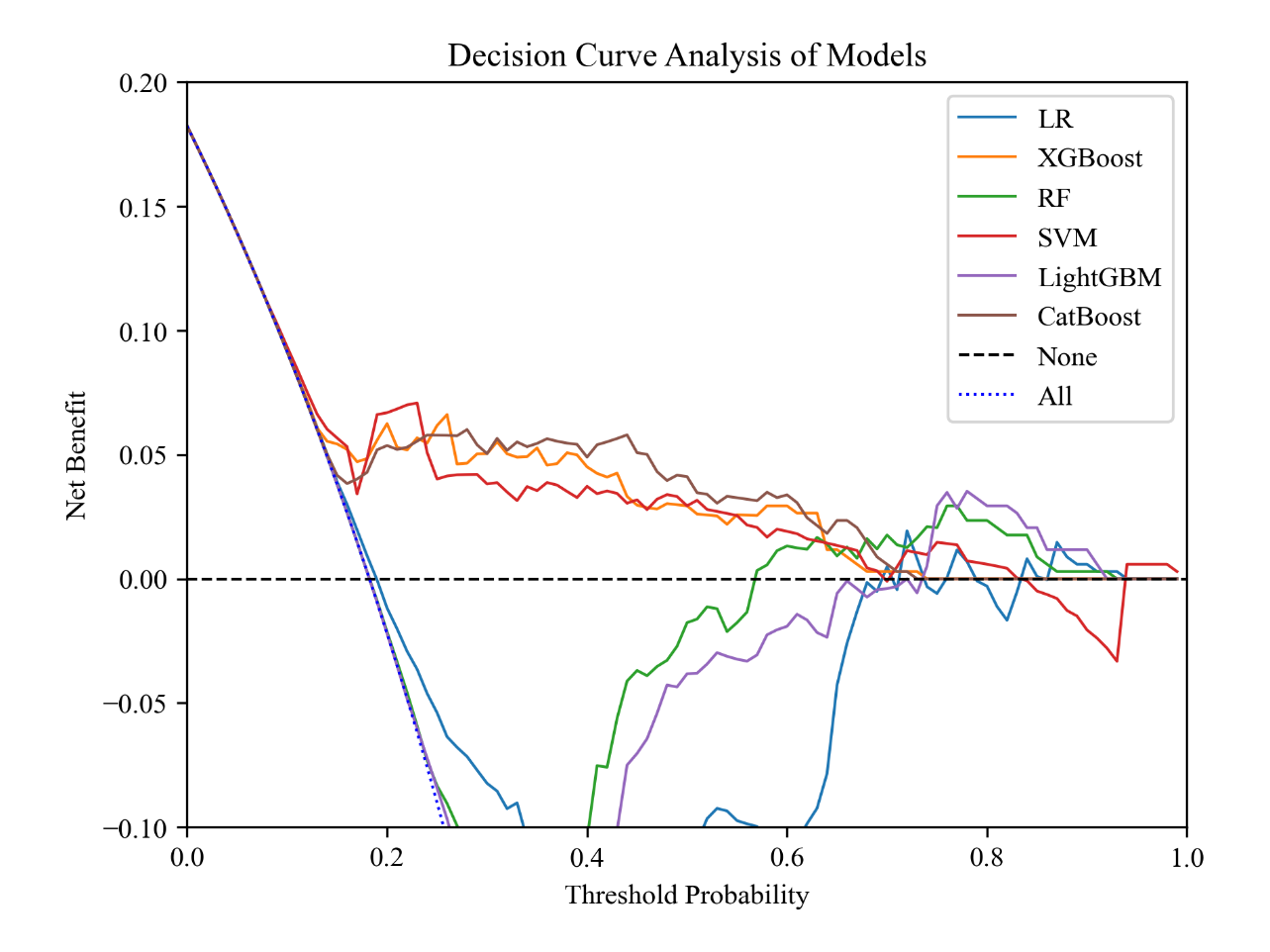


**Supplementary Figure S2.** Decision curve analysis of six machine learning models. **Abbreviations:** LR, logistic regression; XGBoost, extreme gradient boosting; RF, random forest; SVM, support vector machine; LightGBM, light gradient boosting machines; CatBoost, categorical boosting.
